# No-tillage with straw mulching enhances grain yield and water use efficiency of wheat by enhancing sugar metabolism in flag leaves and grains in rainfed Loess Plateau, China

**DOI:** 10.3389/fpls.2026.1817135

**Published:** 2026-05-28

**Authors:** Hutao Wu, Lingling Li, Muhammad Zahid Mumtaz, Haofeng Meng, Yuanhong Zhang, Khuram Shehzad Khan, Zhen Zhu, Xin Tian, Le Wang, Jing Xu

**Affiliations:** 1 College of Agronomy, Gansu Agricultural University, Lanzhou, China; 2 State Key Laboratory of Aridland Crop Science, Gansu Agricultural University, Lanzhou, China; 3 Institute of Molecular Biology and Biotechnology, The University of Lahore, Lahore, Pakistan

**Keywords:** conservation tillage, physiological mechanisms, sucrose metabolism, wheat, yield

## Abstract

**Introduction:**

Tillage and straw management practices play an important role in improving wheat production in rainfed regions. This study aimed to investigate the underlying mechanisms from the perspective of how these practices affect sugar metabolism in flag leaves and grains, as well as the yield formation process of wheat on the Loess Plateau of China.

**Methods:**

This research was conducted in 2023 and 2024 based on a long-term tillage experiment initiated in 2001. The experiment included four treatments: conventional tillage (T), conventional tillage with straw incorporation (TS), no-tillage with no straw (NT), and no-tillage with straw mulching (NTS).

**Results:**

Compared with the T treatment, the NTS treatment significantly increased soil water content by 8% and flag leaf water content (FWC) by 13%. Correlation and path analysis indicated that higher FWC was associated with enhanced activities of flag leaf sucrose phosphate synthase and flag leaf sucrose synthase. Concurrently, flag leaf sucrose content increased, and sucrose was efficiently transported to grains. In the grains, enhanced sucrose synthase activity was linked to increased sucrose cleavage and starch biosynthesis. Consequently, compared with the T treatment, the NTS treatment increased grain yield by 44% and water use efficiency (WUE) by 45%.

**Discussion:**

This study demonstrates that NTS treatment can enhance grain yield and WUE by improving soil water status, optimizing flag leaf water status, and regulating sugar metabolism in flag leaves and grains. This highlights sugar metabolism as an important physiological link between conservation practices and improved wheat productivity in rainfed systems.

## 1 Introduction

Wheat is one of the staple crops in the Loess Plateau, China (Wang et al., 2022). It is crucial to ensure sustainable wheat production for food security in this region (Wu et al., 2023). However, wheat production in this rainfed region is constrained by low and unevenly distributed rainfall, which limits soil water availability, reduces grain yield, and decreases water use efficiency (WUE) (Khan et al., 2020). Conservation tillage practices have been widely reported to improve soil water conditions and crop performance. By reducing soil disturbance and surface evaporation, these practices can enhance soil water retention, reduce runoff and erosion, and improve WUE and yield (Souissi et al., 2020; Chouter et al., 2022; Iqbal et al., 2024). It is evident that conservation tillage contributes to wheat yields by improving soil water conditions.

Carbohydrates are the primary products of photosynthesis, which directly influence crop yield and WUE (Liang et al., 2019). Photosynthetic products of flag leaves contribute approximately 41%–43% of the carbohydrates required for wheat grain filling (Sharma et al., 2003). Wheat flag leaves synthesize carbohydrates via photosynthesis, and these carbohydrates are transported to grains as sucrose. Sucrose is the primary transport form of carbohydrates in wheat. It also acts as a signaling molecule that regulates physiological and metabolic activities (Jiang et al., 2003). The flag leaf sucrose content (FSC) reflects the supply capacity of photosynthetic products to the grains. Higher sucrose levels correspond to an increased supply of photosynthates, which benefits grain filling and yield enhancement (Vimolmangkang et al., 2016). Sucrose phosphate synthase (SPS) and sucrose synthase (SS) play crucial roles in sucrose metabolism in wheat. SPS catalyzes sucrose synthesis in flag leaves and grains, while SS functions in sucrose synthesis and breakdown (Stein and Granot, 2019). SS promotes sucrose synthesis in flag leaves, which is transported via the phloem to the grains (Padhi et al., 2019; Sun et al., 2022). In grains, SS catalyzes the breakdown of sucrose into uridine diphosphate glucose and fructose. These are subsequently used for starch synthesis (Volpicella et al., 2016). Sucrose plays a critical role in carbohydrate transport and yield formation. Therefore, investigating sucrose metabolism in wheat under different tillage practices is crucial for understanding the mechanisms of yield formation and improving WUE.

Numerous studies have examined sucrose metabolism in wheat flag leaves and grains (Zhang et al., 2019a; Ahmed et al., 2020). Drought conditions have been shown to reduce the activity of key enzymes involved in converting sucrose into starch, leading to lower grain starch content (SC) and ultimately decreasing grain yield (Lu et al., 2019). This suggests that external factors, particularly soil water content, strongly influence wheat sucrose metabolism (Zhang et al., 2019a; Ahmed et al., 2020). Although conservation tillage is known to improve soil moisture, wheat productivity and WUE, how it affects sucrose metabolism in flag leaves and grains, and how these changes are associated with grain filling and yield formation, remain insufficiently understood.

To address these knowledge gaps, we leveraged a long-term conservation tillage field experiment established in 2001 in Dingxi City, Gansu Province, and formulated the following hypotheses: (1) Long-term NTS can improve soil water content (SWC) and flag leaf water content (FWC). (2) The increased FWC will enhance the activities of flag leaf sucrose phosphate synthase (FSPS) and flag leaf sucrose synthase (FSS), thereby promoting sucrose synthesis and its translocation to grains. (3) In grains, the elevated SS activity facilitates sucrose cleavage into substrates for starch biosynthesis, accelerating grain filling and consequently increasing grain yield and WUE. (4) Sugar metabolism serves as a key physiological link between soil water conservation and yield improvement. The specific objectives of this study are: (1) To evaluate the effects of different tillage and straw management practices on SWC, FWC, grian yield, and WUE in wheat. (2) To quantitatively analyze the activities of key sucrose-metabolizing enzymes, as well as the contents of soluble sugars, sucrose, and starch in flag leaves and grains. (3) To elucidate how conservation tillage improves wheat grian yield and WUE through sugar metabolism in flag leaves and grains. Aims to provide a scientific basis for optimizing wheat production in rainfed regions of the Loess Plateau.

## 2 Materials and methods

### 2.1 Experimental site

**Figure 1:**
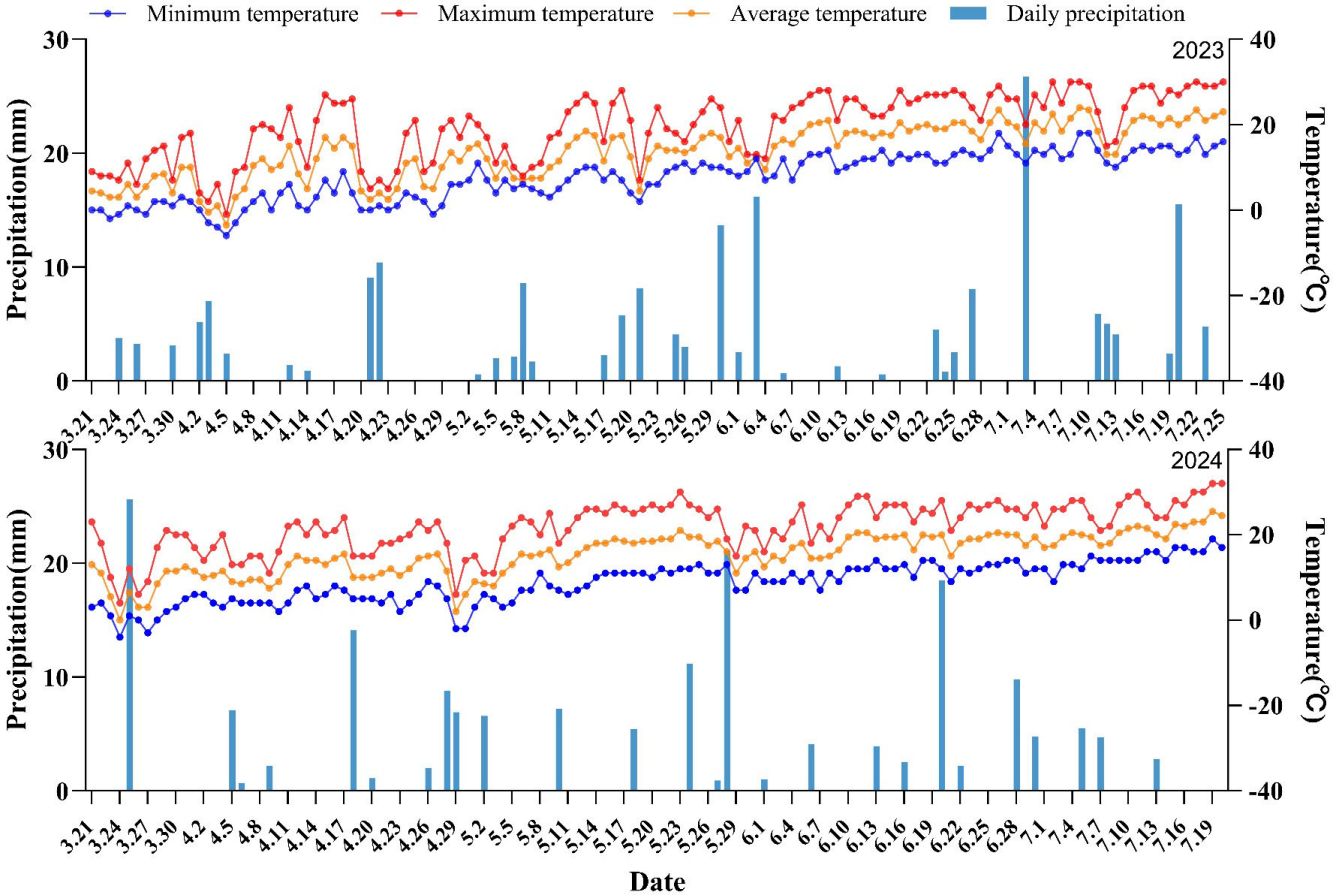
Daily precipitation and air temperature during the wheat growth period in 2023 and 2024 of the experimental area.

The study was conducted at the Experimental Station of Gansu Agricultural University in Lijiabao Town, Dingxi City, Gansu Province, China (104°44′E, 35°28′N). This research was conducted from March 2023 to July 2024, based on a long-term conservation tillage experiment initiated in 2001. The experimental site is situated in the semi-arid region of the Loess Plateau, representing a typical semi-arid rain-fed agricultural zone. The average altitude was 1971 m, with an average annual solar radiation of 593 KJ cm^-2^ and a sunshine duration of 2476 hours. The average annual temperature was 6.7 °C, with accumulated temperatures above 0 °C of 2934 °C, and an accumulated temperature above 10 °C of 2239 °C. The frost-free period lasted for 140 days. The average annual rainfall was 391 mm with a coefficient of variation of 24.3%, while annual evaporation was 1531 mm, and the aridity index was 2.53. The soil was characterized as a typical loess soil, with a soft texture, deep soil layer, uniform texture, and good water-holding capacity. The main physical and chemical properties of the soil are presented in **Table 1**. The rainfall recorded during the growth periods of wheat in 2023 and 2024 was 200.1 mm and 180.7 mm, respectively. **Figure 1** shows the precipitation distribution and temperature variations during the growth period.

**Table 1:** Physicochemical properties of the experimental field soil in 2023.

Treatment	Soil layer (cm)	Bulk density (g cm^-3^)	Porosity (%)	PH	Organic carbon (g kg^-1^)	Total nitrogen (g kg^-1^)	Available phosphorus (mg kg^-1^)	Available potassium (mg kg^-1^)
T	0-5	1.29a	51.70b	8.33a	7.15c	0.86b	12.8b	352.5b
	5-10	1.31a	52.42b	8.35a	6.92c	0.87b	13.1b	329.6a
	10-30	1.32a	51.82b	8.32a	6.44b	0.83c	6.1b	264.3b
TS	0-5	1.23ab	53.66ab	8.32a	8.76b	0.87b	13.4b	364.6a
	5-10	1.26a	52.49b	8.36a	8.22ab	0.88b	13.1b	330.8a
	10-30	1.28a	51.52b	8.31a	7.54ab	0.86b	6.1ab	245.6b
NT	0-5	1.20b	54.63a	8.27b	7.91bc	0.85b	13.2b	348.5b
	5-10	1.22ab	53.82ab	8.30ab	7.47bc	0.89b	11.6c	329.5a
	10-30	1.27a	52.13b	8.26ab	7.00ab	0.85b	5.2b	244.6b
NTS	0-5	1.18b	55.37a	8.23b	9.82a	0.93a	15.6a	367.3a
	5-10	1.16b	56.07a	8.26b	8.91a	0.92a	14.1a	342.5a
	10-30	1.21b	54.40a	8.23b	8.06a	0.93a	7.2a	281.3a

Different lowercase letters in the same column indicate significant differences among treatments (P < 0.05).

### 2.2 Experimental design and crop management

The experiment adopted a two-factor randomized block design, comprising two treatment factors: tillage practice (conventional tillage and no-tillage) and straw management (no straw and straw incorporation/mulching). A total of 4 treatment combinations were set up, with each treatment replicated 3 times. The four treatments are, 1) Conventional tillage (T), 2) Conventional tillage with straw incorporation (TS), 3) No-tillage with no straw (NT), and 4) No-tillage with straw mulching (NTS). Treatment details are described in **Table 2**. Each plot was 80 m² (4 m × 20 m) with 20 cm row spacing. Wheat (cv. Dingxi No. 40) was rotated annually with field pea; this study focused on wheat. Seeding rate was 187.5 kg ha^-^¹. Each treatment received 105 kg ha^-1^ of nitrogen (equivalent to 226.29 kg of urea) and 105 kg ha^-1^ of phosphorus pentoxide (equivalent to 656.25 kg of superphosphate). Wheat was sown in mid−March and harvested in late July.

**Table 2:** Experimental treatments and code descriptions.

Code	Treatment	Specific operation
T	Traditional Tillage	Three-tilled and two-harrowed after harvesting the previous crop and before soil freezing
TS	Traditional Tillage with Straw Incorporation	The operation was the same as T, but all previous crop straw was turned into the soil at the 1st plowing
NT	No-Tillage with No Straw	No tillage all year round, no-till planter applies fertilizer and seeds in one go at planting time
NTS	No-Tillage with Straw Mulching	The operation was the same as NT, and after threshing and harvesting all the crop straw was mulched in the original plots

### 2.3 Measurement items and methods

#### 2.3.1 Determination of soil water content and WUE

Soil samples were collected from five randomly selected points within each plot using a soil auger. The five subsamples from the same soil layer were mixed to form one composite sample per layer. The study focused on the 0–30 cm layer (subdivided into 0–5, 5–10, and 10–30 cm), which represents the primary root distribution zone, with sampling conducted at 0, 7, 14, 21, and 28 days after anthesis. Additionally, soil samples were collected at sowing and harvest from depths of 0–200 cm (divided into 0–5, 5–10, 10–30, 30–50, 50–80, 80–110, 110–140, 140–170, and 170–200 cm). These deep soil samples were used solely for calculating water consumption (ET) and WUE, and not for treatment comparisons. The collected soil samples were oven−dried to determine soil mass water content (Zhang et al., 2019b). SWC was calculated by multiplying mass water content by bulk density. Soil physicochemical properties were measured using the methods described by Mao and Xu (2023). ET and WUE were calculated using Equations 1 and 2 provided by Wang et al. (2018).

(1) water consumption:ET=P+W1−W2

where ET refers to the water consumption (mm). P refers to the precipitation (mm) during the wheat growth period. W1 and W2 refer to the water storage (mm) in the soil layer within 0–200 cm before wheat sowing and after wheat harvesting.

(2) $$\text{Water use efficiency}:WUE=\frac{GY}{ET}$$

WUE stands for water use efficiency (kg ha^-1^ mm^-^¹). GY represents grain yield (kg ha^-1^). ET represents water consumption (mm).

#### 2.3.2 Determination of sugar content and key sucrose-metabolizing enzyme activities in flag leaves and grains

During the flowering period, blooming wheat plants exhibiting uniform growth were selected. The single stems from 100 wheat plants in each plot were marked. At 0, 7, 14, 21, and 28 days after anthesis, twenty flag leaves and ten spikes (with 1–2 grains taken from the middle of each spike) were collected from each plot. These samples were divided into two parts: one part consisting of flag leaves and grains, which were weighed immediately to obtain the fresh weight after sampling. Subsequently, the samples were fixed at 105 °C and dried at 70 °C to determine the dry weight, after which the flag leaf water content (FWC) was calculated according to Equation 3:

(3) $$FWC(\%)=\frac{FFW-FDW}{FFW}\times 100\%$$

FFW represents flag leaf fresh weight (g). FDW represents flag leaf dry weight (g).

The dried samples from the first part were used to determine soluble sugar, sucrose, and starch contents. The second part of the samples was stored at -80 °C for sucrose metabolic enzyme activity assays. Soluble sugar and starch contents were measured using the method of Bader et al. (2024). Sucrose content was determined according to the method of Wei et al. (2023). The activities of sucrose phosphate synthase (SPS) and sucrose synthase (SS) were assessed following the methods outlined by Kalwade and Devarumath (2014).

#### 2.3.3 Richards model and its characteristic parameters

The dynamics of grain filling were fitted with the Richards growth equation and calculated using Equation 4 provided by Liang et al. (2024):

(4) $$W=\frac{A}{{\left(1+Be^{-kt}\right)}^{1/N}}$$

The grain filling rate R (mg grain^-1^ d^-1^) was obtained by taking the derivative of the Equation 5:

(5) $$R=\frac{AkBe^{-kt}}{N{\left(1+Be^{-kt}\right)}^{(N+1)/N}}$$

In this equation, W is grain mass at time t, A is theoretical maximum grain mass, B is the initial growth parameter, k is the growth rate parameter, t is days after anthesis, and N is the curve shape parameter. When 0 < N < 1, filling is strongly limited by storage capacity. When N > 1, storage capacity has little limitation on filling.

#### 2.3.4 Determination of grain yield and its components

During the maturity stage of wheat, 1 m² of wheat was randomly selected from each plot for spike count. In each experimental plot, 20 plants were randomly sampled to determine grains per spike. The thousand-grain weight for each experimental plot was measured. Subsequently, manual harvesting was performed from the central area of each plot after excluding a 50 cm border, at a cutting height of 5 cm above the ground. Following the assessment of biological yield, the grains were threshed, and the grain yield per plot was calculated after drying to a grain water content of 130 g kg^-^¹.

### 2.4 Data analysis

Data were organized and summarized using Microsoft Excel 2019. Statistical analysis was performed using SPSS 25.0 software (IBM Corporation, Chicago, USA). The tillage effect (T) and straw effect (S) were set as fixed factors, and the repeated experiments were set as random effects. The sugar metabolism-related indicators of wheat flag leaves and grains under different tillage measures were evaluated using two−factor analysis of variance (ANOVA) with Duncan’s multiple range test for comparisons at P ≤ 0.05. Data figures were drawn using GraphPad Prism 10. Correlation analysis was performed using the Pearson correlation coefficient, and a correlation heatmap was generated using the web-based tool Chiplot. Partial least squares regression (PLS) analysis was conducted using SPSS 25.0, and then a path analysis diagram was created using Microsoft PowerPoint.

## 3 Results

### 3.1 Effects of different tillage practices on soil physicochemical properties

After 22 years of continuous implementation of different treatments, significant differences in soil physicochemical properties were observed among the treatments (**Table 1**). Compared with the T treatment, both no-tillage treatments (NT and NTS) and straw treatments (TS and NTS) increased soil porosity, soil organic carbon (SOC), and total nitrogen (TN). These increases occurred in the 0–5 cm, 5–10 cm, and 10–30 cm soil layers. Compared with the T treatment, the NTS treatment significantly increased the contents of SOC, TN, available phosphorus (AP), and available potassium (AK). These increases were also observed in the 0–5 cm, 5–10 cm, and 10–30 cm soil layers. In the 0–5 cm and 5–10 cm soil layers, the soil porosity under NTS treatment was 7.10% and 6.96% higher than that under T treatment, respectively.

### 3.2 Effects of different tillage practices on soil water content and flag leaf water content

**Figure 2:**
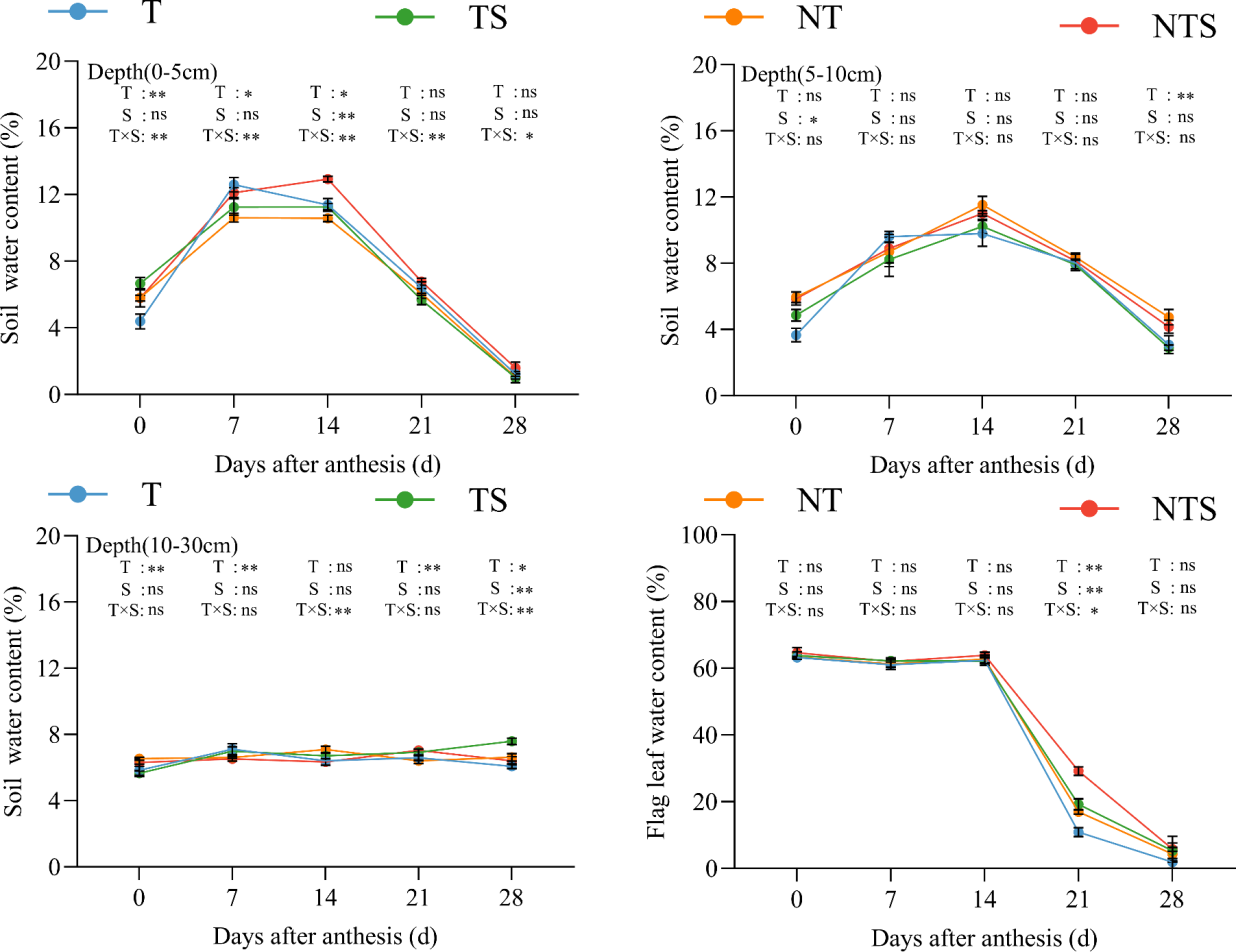
Dynamic changes of soil water content in the 0–30 cm soil layer and flag leaf water content during the growth period after anthesis of wheat in 2024. T, S, and T×S in the figure represent the effects of tillage practice, straw mulching, and their interaction, respectively. * significant; ** highly significant; ns, non-significant.

The SWC in the 0–30 cm layer varied with treatment measures and growth stages (**Figure 2**). Compared with the T treatment, both no-tillage treatments (NT and NTS) and straw treatments (TS and NTS) increased the average SWC. This increase occurred in the 0–30 cm layer during 0 to 28 days after anthesis. At the flowering stage (0 days after anthesis), the NTS treatment significantly increased the SWC compared with the T treatment. In the 0–5 cm layer, the increase was 31.84%. In the 5–10 cm layer, the increase was 59.95%. In the 10–30 cm layer, the increase was 8.06%. At 14 days after anthesis, the SWC under the NTS treatment was higher than that under T treatment in the 0–5 cm and 5–10 cm layers. Specifically, the increases were 13.67% in the 0–5 cm layer and 12.33% in the 5–10 cm layer. By 28 days after anthesis, the SWC in the 5–10 cm layer under NTS treatment was 35.28% higher than that under T treatment.

The FWC across all treatments showed a declining trend at 14 days after anthesis (**Figure 2**). Compared with the T treatment, both no-tillage treatments (NT and NTS) and straw treatments (TS and NTS) increased FWC after anthesis in wheat. The NTS treatment exhibited significantly higher FWC than other treatments at 21 days after anthesis (*P* < 0.01). Compared with the T treatment, NTS, TS, and NT treatments increased the FWC by 168.05%, 76.04%, and 55.95%, respectively (*P* < 0.01).

### 3.3 Effect of different tillage practices on sugar metabolism in flag leaves

#### 3.3.1 Key enzyme activities of sucrose metabolism in flag leaves under different tillage practices

**Figure 3:**
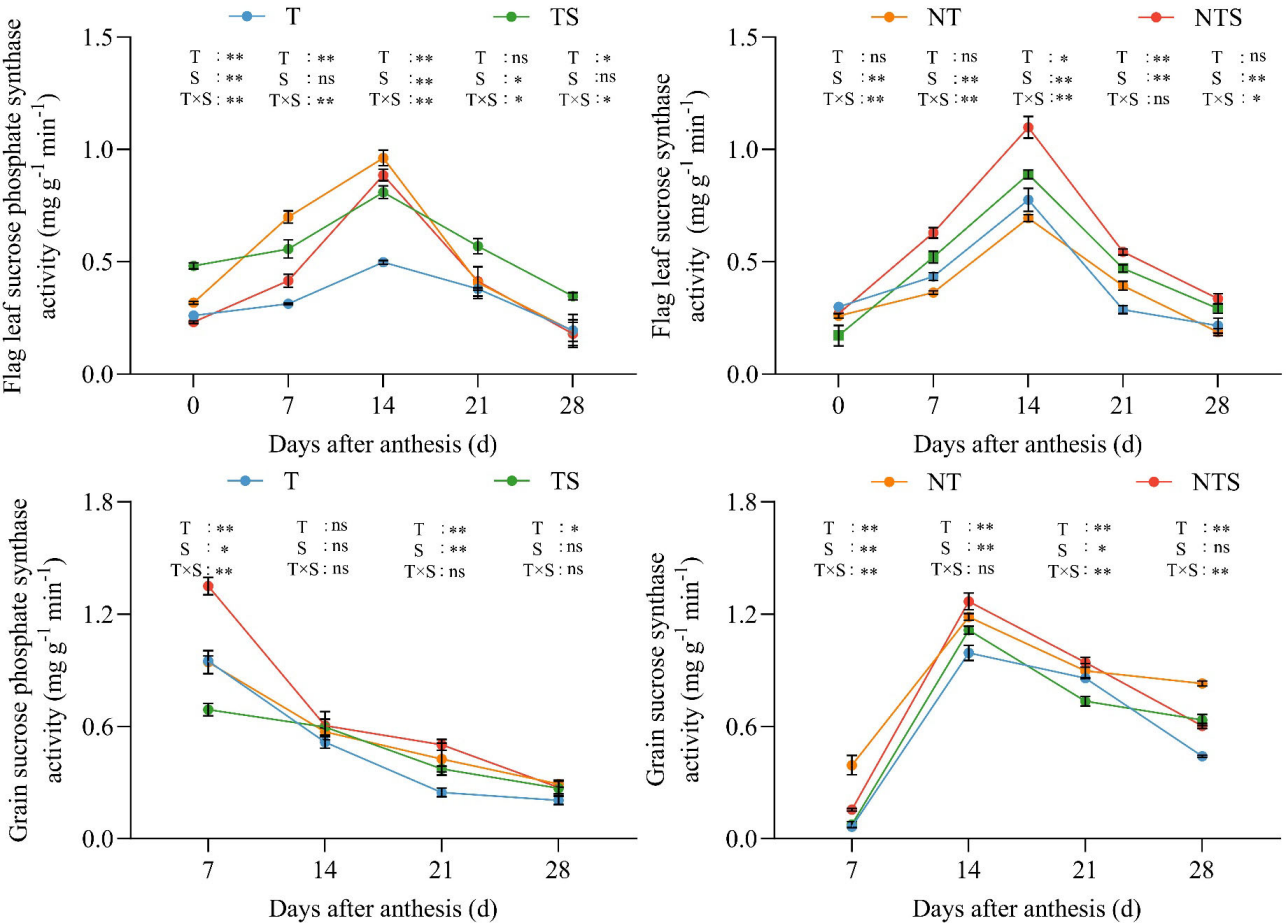
Dynamics of sucrose phosphate synthase (SPS) and sucrose synthase (SS) activities in flag leaves and grains of wheat after anthesis in 2024. T, S, and T×S in the figure represent the effects of tillage practice, straw mulching, and their interaction, respectively. * significant; ** highly significant; ns, non-significant.

The flag leaf sucrose phosphate synthase (FSPS) activity under all treatments reached its peak at 14 days after anthesis (**Figure 3**). Compared with the T treatment, both no-tillage treatments (NT and NTS) and straw treatments (TS and NTS) increased the FSPS activity in wheat during 0 to 21 days after anthesis. During 7 to 14 days after anthesis, the FSPS activity of the TS treatment was 62.58%–78.38% higher than that of the T treatment. The FSPS activity of the NTS treatment was 93.22%–123.56% higher. The FSPS activity of the NT treatment was 33.86%–77.93% higher.

The flag leaf sucrose synthase (FSS) activity also peaked at 14 days after anthesis (**Figure 3**). Compared with the T treatment, both no-tillage treatments (NT and NTS) and straw treatments (TS and NTS) increased the FSS activity in wheat. This increase occurred from 7 to 28 days after anthesis. During 7 to 21 days after anthesis, the NTS treatment significantly enhanced FSS activity by 41.66%–89.70% compared with the T treatment.

#### 3.3.2 Flag leaf soluble sugar content under different tillage practices

**Figure 4:**
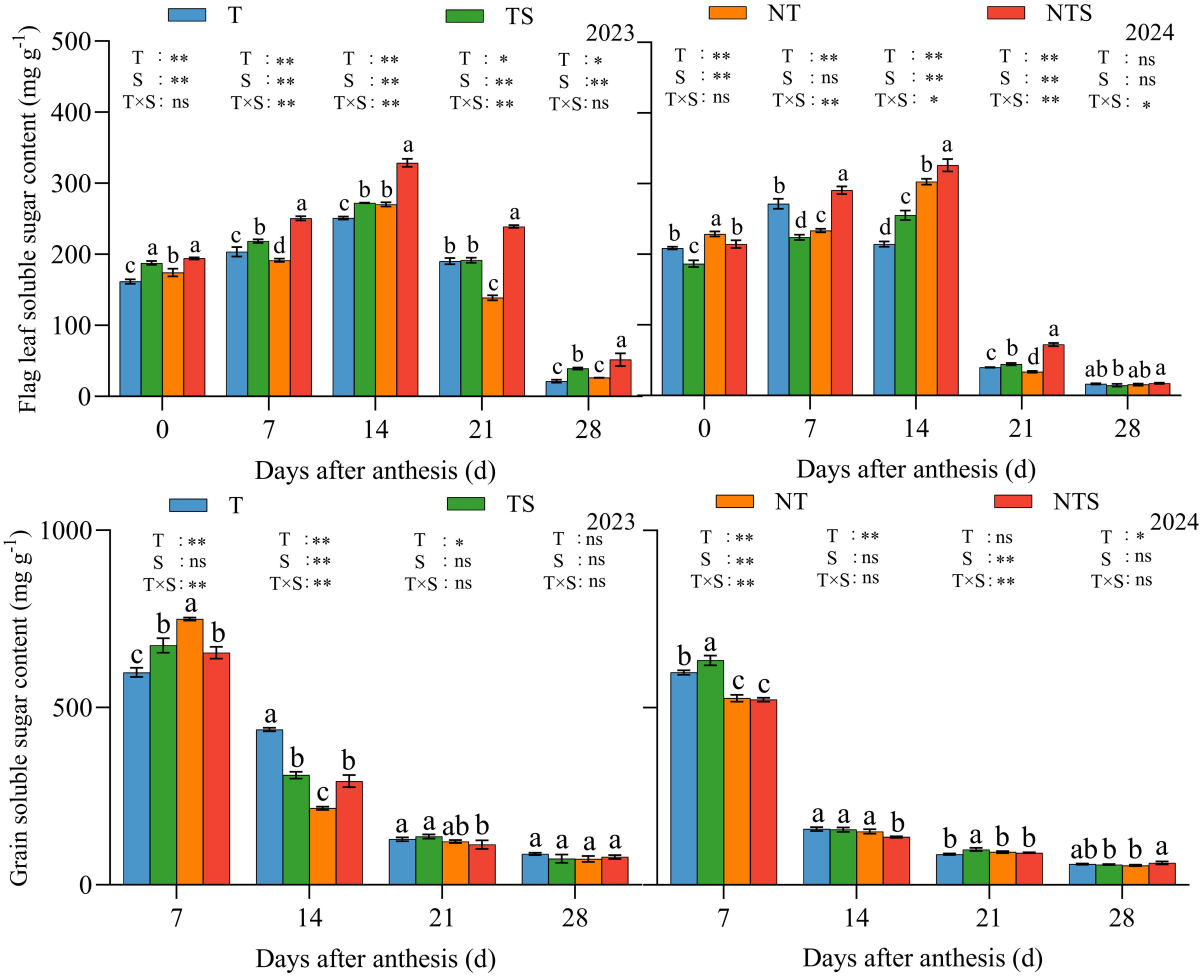
Dynamics of soluble sugar content in flag leaves and grains of wheat after anthesis in 2023 and 2024. T, S, and T×S in the figure represent the effects of tillage practice, straw mulching, and their interaction, respectively. * significant; ** highly significant; ns, non-significant.

Except for the T treatment in 2024, the FSSC values of all other treatments reached their maximum values at 14 days after anthesis (**Figure 4**). During the two experimental years (2023–2024), compared with the T treatment, both no-tillage treatments (NT and NTS) and straw treatments (TS and NTS) increased the average FSSC during the growth period. From 7 to 21 days after anthesis in 2023 and 2024, the NTS treatment increased the FSSC by 23.11%–30.90% in 2023 and by 7.04%–81.42% in 2024, compared with the T treatment.

#### 3.3.3 Flag leaf sucrose content under different tillage practices

**Figure 5:**
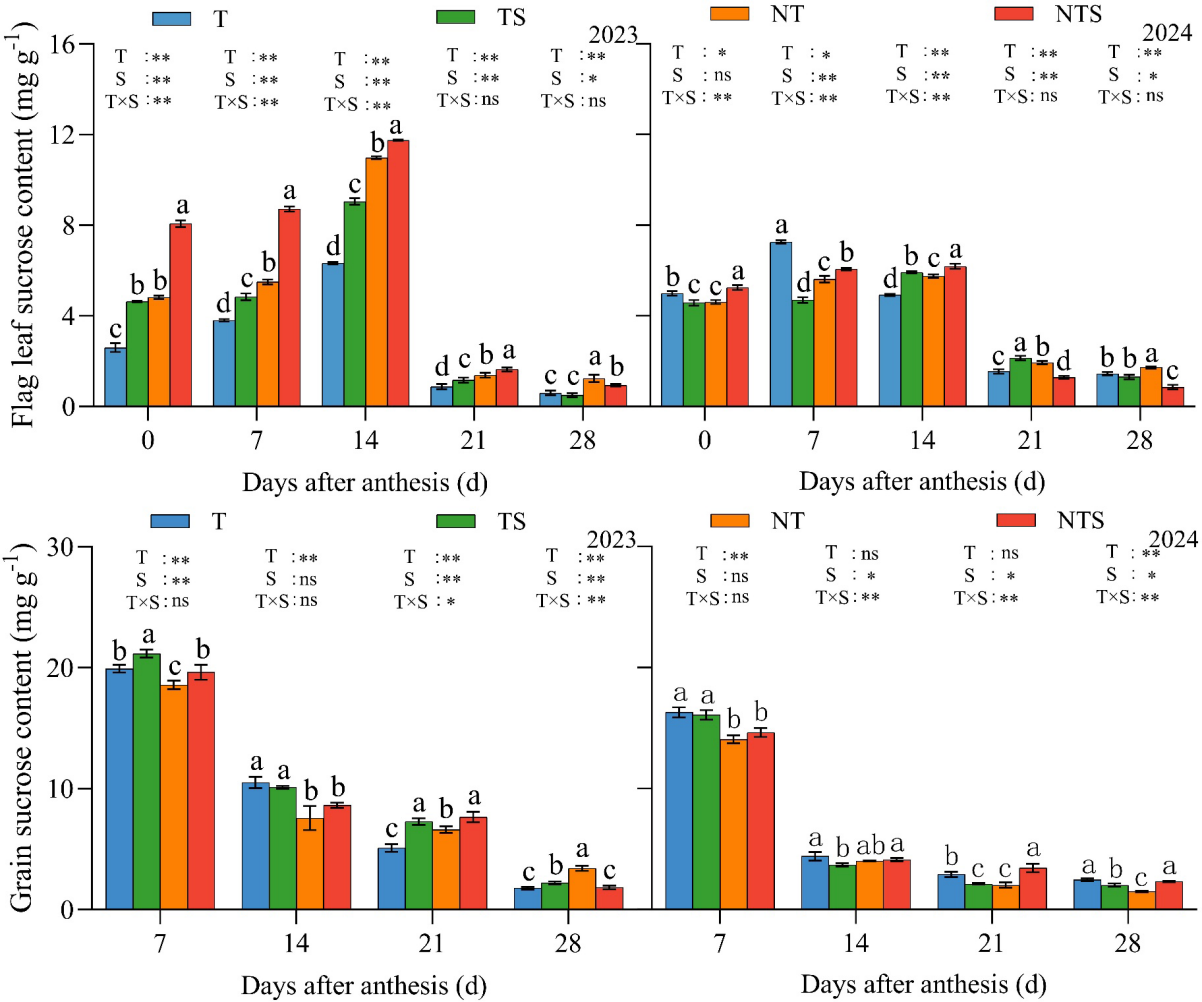
Dynamics of sucrose content in flag leaves and grains of wheat after anthesis in 2023 and 2024. T, S, and T×S in the figure represent the effects of tillage practice, straw mulching, and their interaction, respectively. * significant; ** highly significant; ns, non-significant.

Except for the T treatment in 2024, the FSC also peaked at 14 days after anthesis under other treatments (**Figure 5**). During the two experimental years (2023–2024), compared with the T treatment, both no-tillage treatments (NT and NTS) and straw treatments (TS and NTS) increased the FSC of wheat. This increase occurred from 14 to 21 days after anthesis. Data from both years showed that the average FSC under NTS treatment was 47.55% higher than that under T treatment. Under NT treatment, it was 26.62% higher. Under TS treatment, it was 12.93% higher. Data from 2023 showed that during 0 to 21 days after anthesis, the FSC in TS treatment was 27.04%–78.28% higher than that in T treatment. In NT treatment, it was 44.28%–85.58% higher. In NTS treatment, it was 85.68%–210.53% higher. Data from 2024 indicated that the FSC under NTS treatment was significantly increased compared with the T treatment. At 0 days after anthesis, the increase was 5.11%. At 14 days after anthesis, the increase was 25.57%.

### 3.4 Effects of different tillage practices on the sugar metabolism of grains

#### 3.4.1 Key enzyme activities of sucrose metabolism in grains under different tillage practices

The grain sucrose phosphate synthase (GSPS) activity gradually decreased after anthesis (**Figure 3**). Compared with the T treatment, both no-tillage treatments (NT and NTS) and straw treatments (TS and NTS) increased the GSPS activity. Compared with the T treatment, the NTS treatment increased GSPS activity by 42.13% at 7 days after anthesis and by 102.80% at 21 days after anthesis.

The grain sucrose synthase (GSS) activity exhibited a unimodal change pattern (**Figure 3**). It reached its peak at 14 days after anthesis. Compared with the T treatment, both no-tillage treatments (NT and NTS) and straw treatments (TS and NTS) increased the average GSS activity during the wheat growth period. At 14 days after anthesis, the NTS treatment increased GSS activity by 27.72% compared with the T treatment.

#### 3.4.2 Grain soluble sugar content under different tillage practices

The GSSC gradually decreased after anthesis (**Figure 4**). During the two experimental years (2023–2024), compared with the T treatment, both no-tillage treatments (NT and NTS) and straw treatments (TS and NTS) reduced the average GSSC. Compared with the T treatment, the NT and NTS treatments decreased the average GSSC by 7.86% and 9.52%, respectively.

#### 3.4.3 Grain sucrose content under different tillage practices

The GSC also showed a decreasing trend after anthesis (**Figure 5**). During the two experimental years (2023–2024), compared with the T treatment, no-tillage treatments (NT and NTS) reduced the average GSC in wheat. In both experimental years, the NT treatment decreased the average GSC by 8.83% compared with the T treatment. At 7 days and 14 days after anthesis, the GSC in wheat under NT and NTS treatments was lower than that under the T treatment. At 21 days after anthesis, the GSC under the NTS treatment increased compared with the T treatment. In 2023, the increase was 50.15%. In 2024, the increase was 18.30%.

#### 3.4.4 Grain starch content under different tillage practices

**Figure 6:**
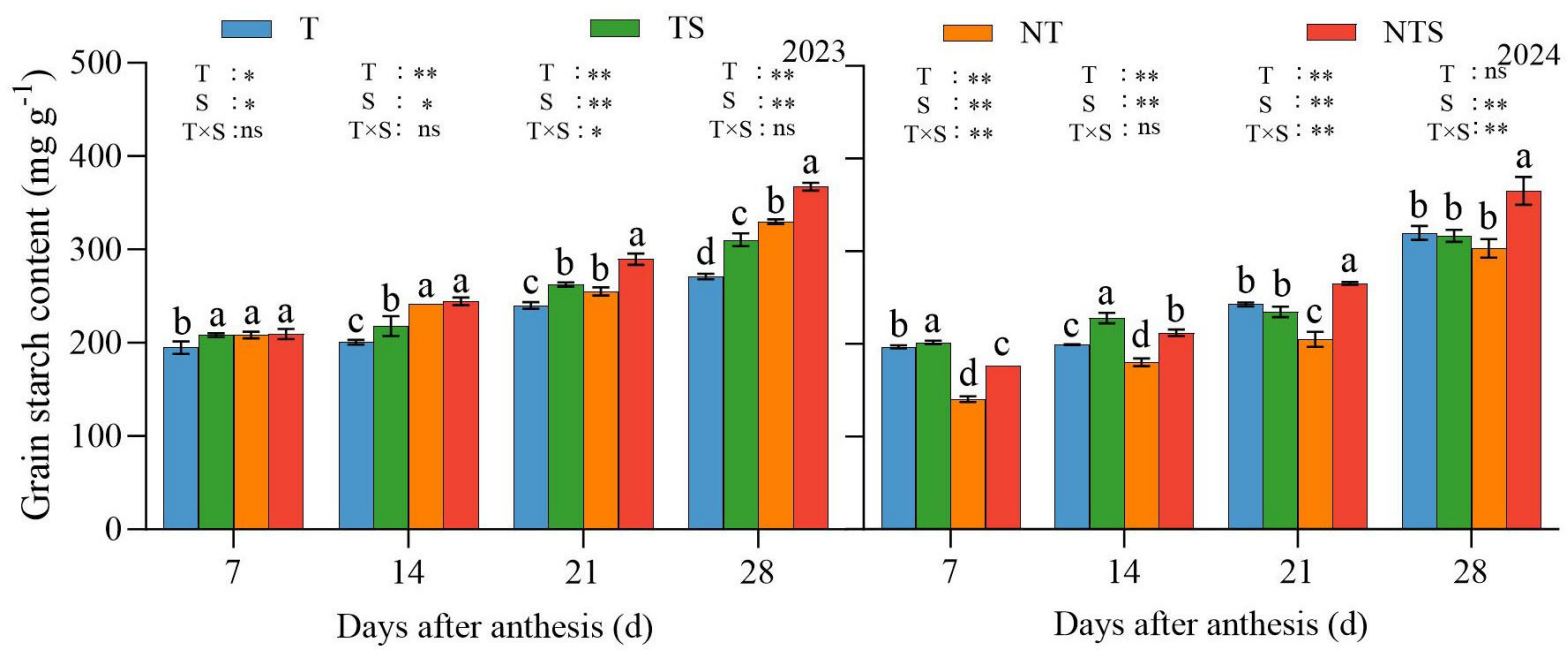
Dynamics of grain starch content in wheat after anthesis in 2023 and 2024. T, S, and T×S in the figure represent the effects of tillage practice, straw mulching, and their interaction, respectively. * significant; ** highly significant; ns, non-significant.

The SC continuously increased during the grain filling period (**Figure 6**). In both experimental years (2023–2024), the straw treatments (TS and NTS) enhanced the average SC in wheat compared with the T treatment. Across the two experimental years, NTS increased the average SC by 14.16% compared with the T treatment. During the 21 to 28 days after anthesis, NTS increased SC by 20.61%–35.35% in 2023 and by 9.25%–14.26% in 2024 compared with the T treatment.

### 3.5 Effects of different tillage practices on grain filling

**Table 3** summarizes grain filling parameters. Compared with the T treatment, both no-tillage treatments (NT and NTS) and straw treatments (TS and NTS) increased the maximum grain filling rate (GRmax) and average grain filling rate (GRmean). Compared with the T treatment, the NTS treatment significantly increased GRmax by 27.01%. It also increased GRmean by 9.85%. In addition, it increased the theoretical maximum grain weight (Wmean) by 4.35%.

**Table 3:** Effects of different tillage practices on grain filling parameters of wheat after anthesis in the 2024 experimental year.

Treatment	R_0_ (mg grain^-1^ d^-1^)	Tmax(d)	GRmax (mg grain^-1^ d^-1^)	GRmean (mg grain^-1^ d^-1^)	Wmean (mg grain^-1^)
T	0.55 ± 0.12a	14.81 ± 0.92a	1.90 ± 0.14b	0.84 ± 0.02c	33.58 ± 0.44b
TS	0.32 ± 0.13a	14.20 ± 0.66a	2.18 ± 0.13ab	0.86 ± 0.03bc	31.93 ± 0.24c
NT	0.46 ± 0.04a	15.30 ± 0.39a	2.16 ± 0.10ab	0.89 ± 0.02ab	34.45 ± 1.29ab
NTS	0.42 ± 0.19a	15.29 ± 0.94a	2.41 ± 0.27a	0.92 ± 0.02a	35.04 ± 0.23a

R_0,_ initial grain filling rate; Tmax, time at maximum grain filling rate; GRmax, maximum grain filling rate; GRmean, average grain filling rate; Wmean, theoretical maximum grain weight. Different lowercase letters in the same column indicate significant differences among treatments (P < 0.05).

### 3.6 Effect of different tillage practices on grain yield and WUE

During the two experimental years (2023–2024), both no-tillage treatments (NT and NTS) and straw treatments (TS and NTS) increased wheat grain yield, spike count, grains per spike, biomass yield, and WUE compared with the T treatment (**Table 4**). In the experimental years 2023 and 2024, compared with the T treatment, the NTS treatment increased grain yield by 35.42%–62.08%. The spike count of wheat increased by 13.00%–41.58%. The grains per spike increased by 15.09%–41.16%. Moreover, WUE increased by 17.32%–72.14%.

**Table 4:** Effect of different tillage practices on grain yield and WUE.

Year	Treatment	Spike count m^-2^	Grains per spike	Thousand-grain weight (g)	Grain yield (kg ha^-1^)	Harvest index (%)	WUE (kg ha^-1^ mm^-1^)
2023	T	291 ± 30b	32 ± 1b	31.42 ± 0.70a	725.32 ± 102.08b	0.35 ± 0.02a	3.25 ± 0.39b
	TS	329 ± 28b	32 ± 2b	31.47 ± 0.58a	969.53 ± 81.07ab	0.37 ± 0.04a	4.24 ± 0.30b
	NT	318 ± 28b	33 ± 2b	30.54 ± 3.75a	921.15 ± 172.21ab	0.38 ± 0.04a	4.29 ± 0.79ab
	NTS	412 ± 32a	37 ± 1a	32.85 ± 3.60a	1175.63 ± 215.34a	0.35 ± 0.02a	5.59 ± 0.86a
2024	T	350 ± 26b	26 ± 4b	33.52 ± 0.42ab	1452.16 ± 126.71b	0.40 ± 0.01a	5.78 ± 0.03b
	TS	405 ± 11a	30 ± 4ab	32.67 ± 0.65b	1537.47 ± 12.65b	0.39 ± 0.02a	6.34 ± 0.26ab
	NT	423 ± 15a	35 ± 3a	33.42 ± 2.15ab	1643.74 ± 180.03b	0.43 ± 0.04a	6.25 ± 0.38bc
	NTS	396 ± 8a	37 ± 5a	35.16 ± 0.31a	1966.49 ± 251.55a	0.41 ± 0.02a	6.78 ± 0.38a
2023	T(tillage)	*	*	ns	ns	ns	*
	S(straw mulching)	*	ns	ns	*	ns	**
	T×S	ns	ns	ns	ns	ns	ns
2024	T(tillage)	*	*	ns	*	ns	ns
	S(straw mulching)	ns	ns	ns	ns	ns	**
	T×S	**	ns	ns	ns	ns	*
	Y(years)	**	ns	*	**	**	*

WUE, water use efficiency. T, S, and T×S in the figure represent the effects of tillage practice, straw mulching, and their interaction, respectively. Different lowercase letters in the same column indicate significant differences among treatments (P < 0.05). * significant; ** highly significant; ns, non-significant.

### 3.7 Correlation and path analysis between different tillage practices and SWC, sucrose metabolism parameters in flag leaves and grains, grain yield and WUE

**Figure 7:**
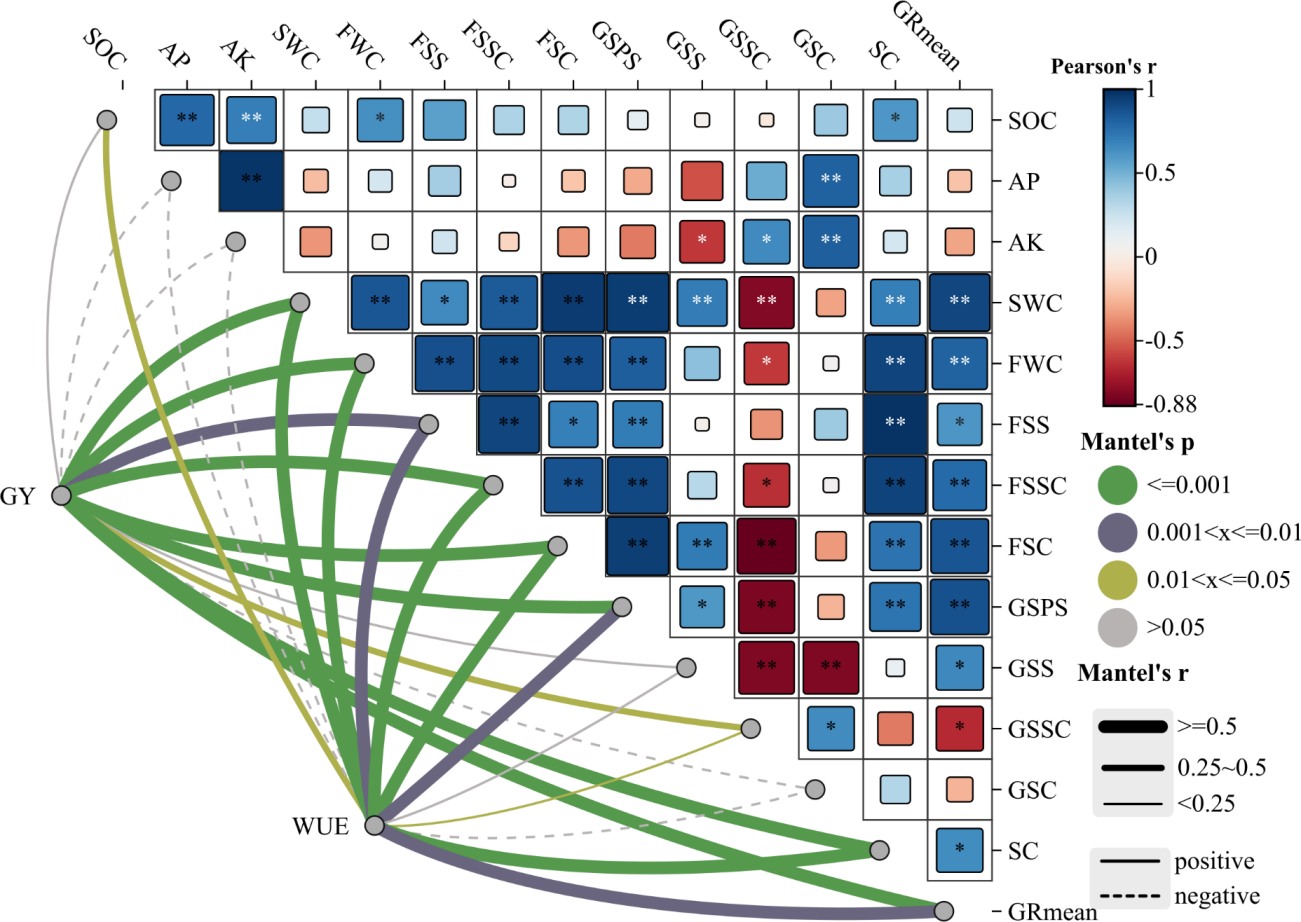
Correlation analysis of soil water content, sucrose metabolism parameters in flag leaves and grains, grain yield and water use efficiency in 2023 and 2024. SOC, AP, AK, SWC, FWC, FSS, FSSC, FSC, GSPS, GSS, GSSC, GSC, SC, GRmean, GY and WUE denote soil organic carbon, available phosphorus, available potassium, soil water content, flag leaf water content, flag leaf sucrose synthase, flag leaf soluble sugar content, flag leaf sucrose content, grain sucrose phosphate synthase, grain sucrose synthase, grain soluble sugar content, grain sucrose content, grain starch content, average grain filling rate, grain yield, and water use efficiency, respectively. * indicates correlation, ** indicates strong correlation.

The detailed correlations between sucrose metabolism indicators in flag leaves and grains are shown in **Figure 7**. All sucrose metabolism indicators showed positive correlations with SWC and FWC (except GSSC and GSC). The GRmean exhibited significant positive correlations with FSS, FSSC, FSC, GSPS, GSS, and SC (*P* < 0.05). Both grain yield and WUE were significantly positively correlated with SWC, FWC, FSS, FSSC, FSC, GSPS, SC, and GRmean (*P* < 0.05).

**Figure 8:**
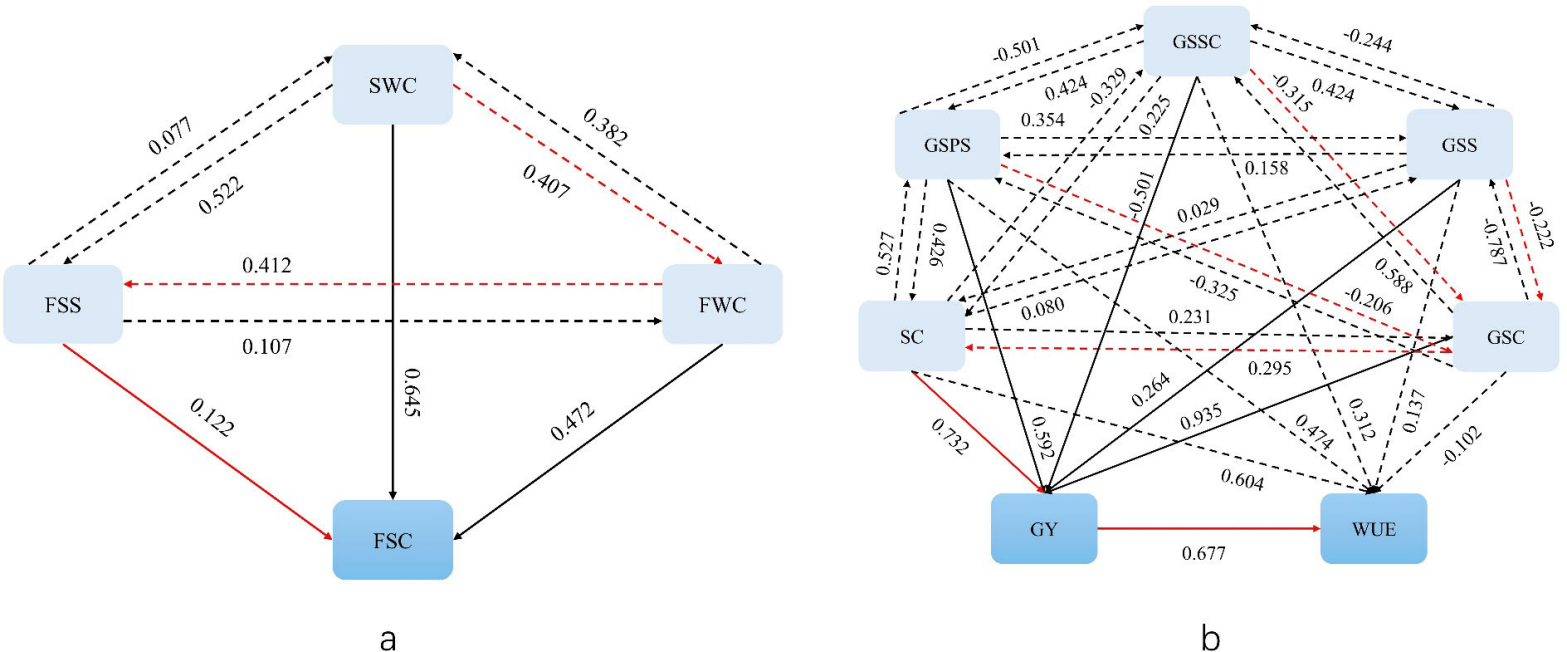
Partial least squares regression (PLS). a represents the flag leaf, b represents the grain. Solid lines indicate direct regression relationships between the indicators, while dashed lines represent indirect regression relationships. Numbers denote regression coefficients. The red bold line segments and red numbers represent the main paths and regression coefficients through which each parameter affects grain yield, respectively. SWC, FWC, FSS, FSC, GSPS, GSS, GSSC, GSC, SC, GY, and WUE represent soil water content, flag leaf water content, flag leaf sucrose synthase, flag leaf sucrose content, grain sucrose phosphate synthase, grain sucrose synthase, grain soluble sugar content, grain sucrose content, grain starch content, grain yield, and water use efficiency, respectively.

This study employed partial least squares regression analysis (**Figure 8**). In the flag leaf, the direct regression coefficients of SWC, FWC, and FSS with FSC were ranked in descending order. SWC had the highest coefficient (0.645), followed by FWC (0.472) and FSS (0.122), indicating that SWC contributed the most to FSC. The indirect regression coefficients revealed that SWC primarily influenced FSC indirectly through FWC and FSS. This ultimately enhanced FSC and facilitated sucrose translocation to the grains. In grains, the direct regression coefficients of sugar metabolism parameters for grain yield were ranked as follows: GSC (0.935) > SC (0.732) > GSPS (0.592) > GSSC (-0.501) > GSS (0.264). Thus, GSC contributed the most to grain yield. Regarding the indirect regression coefficients for grain yield, it can be seen that GSPS, GSSC, and GSS mainly affected grain yield indirectly through GSC, while GSC in turn indirectly influenced grain yield through SC. Ultimately, grain yield directly affected WUE.

## 4 Discussion

### 4.1 NTS enhanced grain yield and WUE

Water deficiency in the soil limits the growth and development of wheat by affecting photosynthesis, which ultimately has an adverse impact on grain yield (Rubén et al., 2018). Conservation tillage can promote water uptake in wheat by increasing water retention in soil (Lamptey et al., 2020). In this study, the NTS treatment significantly increased soil water content in the 0–10 cm layer of wheat during 14 to 21 days after anthesis (**Figure 2**). This increase in soil water content also contributed to enhanced FWC in NTS-treated wheat during the same period (**Figure 2**). The increase in soil water content may be partly attributed to the minimal soil disturbance associated with no-tillage practices. These practices promote the formation of soil aggregates and enhance porosity, thereby creating biological pores that improve water infiltration and permeability (Li et al., 2025a). The NTS technique preserves soil water by blocking solar radiation and wind erosion on the soil surface, thereby preventing water evaporation loss (Zhao et al., 2019). The straw cover further leads to organic matter accumulation in soil, which improves soil aggregation, leading to better water-holding capacity (Yang et al., 2023). Stagnari et al. (2014) reported an increase in soil water content through conservation tillage, which promoted water uptake by roots through osmotic absorption (Sharma et al., 2010).

This study revealed higher water contents in flag leaves of wheat after anthesis under NTS treatment (**Figure 2**). This might be attributed to higher water retention in soil, which in turn promotes WUE, leaf hydration, and osmotic adjustment. The increased FWC may contribute to enhancing the net photosynthetic rate and the transport of photosynthetic products, thereby promoting grain yield (Li et al., 2025b). The increase in grain yield might be due to higher leaf water contents and WUE under NTS treatment (**Table 4**). These results were consistent with the findings of Kaur and Mahal (2017) and Wu et al. (2017). Wardlaw and Willenbrink, 2000 and Wardlaw (2002) demonstrated that FWC is crucial for sustaining the production of photosynthetic products during critical growth stages, which ultimately influences grain yield. A strong positive correlation was observed between FWC and grain yield, indicating a significant impact of FWC on grain yield (**Figure 7**). This correlation may be due to several interrelated physiological and biochemical mechanisms. To explore the potential underlying causes at the biochemical level, we subsequently investigated the effects of NTS on sugar metabolism. At the grain filling stage in wheat, the flag leaf serves as a primary source of photosynthates. It plays a crucial role in grain filling and thereby contributes to increased grain yield and WUE (Biswal and Kohli, 2013). Under NTS treatment, the higher FWC might sustain photosynthesis by maintaining turgor pressure and cellular hydration (Alsamin et al., 2022). The enhancement of photosynthesis facilitates the accumulation of FSSC. Additionally, the increased FWC enhances FSS activity, promoting sucrose accumulation in these leaves. This process is conducive to the efficient transport of sucrose to the grains, ultimately increasing grain yield and WUE (Ba et al., 2020; Ma et al., 2021).

In summary, the NTS treatment significantly increased the soil water content in the 0–10 cm layer from 14 to 21 days after anthesis by reducing soil disturbance and increasing surface coverage. The improved soil water conditions further enhanced flag leaf water status, these water improvements enhance grain yield, ultimately enhances WUE.

### 4.2 NTS optimizes sugar metabolism in flag leaves and grains by improving SWC and FWC, thereby increasing grain yield

The flag leaf is considered the primary source of carbohydrates during grain filling, and its sugar metabolism is significantly influenced by environmental factors (Biswal and Kohli, 2013). These metabolic processes are regulated by SPS and SS through the synthesis, transport, and conversion of sucrose (Jiang et al., 2022). In this study, the NTS treatment significantly increased SPS and SS activities in the flag leaves and grains of wheat (**Figure 3**). This increase is primarily attributed to the enhanced water-holding capacity of the soil. This capacity promotes FWC, which in turn improves the activity of FSPS and FSS enzymes. Consequently, sucrose levels in the flag leaf are ultimately increased. In addition, higher water content in the flag leaf supports the transport of sucrose from the leaf to the grains (Durand et al., 2016; Verma et al., 2019; Barbosa et al., 2023). The increase in FSPS activity may regulate sucrose biosynthesis in the flag leaf, thereby facilitating a more efficient carbohydrate supply for grain filling (Ma et al., 2024). SS is also involved in the catabolism of sucrose in grains, contributing to increased starch accumulation and grain yield (Cabello et al., 2014). The increased FSC during the mid-stage of grain filling ensures adequate sucrose supply to the grains, which is advantageous for starch accumulation and overall grain yield enhancement (Fan et al., 2022). TN, AP, and AK in the soil are crucial factors influencing wheat yield (Wang et al., 2018a). These nutrients are vital for grain development. Nitrogen plays a critical role in protein synthesis and photosynthetic activity. Phosphorus plays a significant role in energy transfer and root development (Bouain et al., 2016; Fu et al., 2022). Potassium regulates enzyme activity, water balance, and transport of carbohydrates (Armengaud et al., 2009).

The study showed that after the flowering stage, the FSSC and FSC under NTS treatment significantly increased, thereby enhancing grain filling (**Figures 4**, **5**). This treatment also increased the SC, which was associated with the FWC. Furthermore, the activities of GSPS and GSS had a significant direct impact on grain filling (**Figures 6**, **7**). In addition, the FWC indirectly influenced grain yield by affecting FSS activity and FSC (**Figure 8**). Similarly, GSS indirectly affected grain yield through its impact on GSC and SC (**Figure 8**). Moreover, this treatment significantly increased the levels of SOC, TN, AP, and AK in the soil (**Table 1**). Notably, AP and AK exhibited significant positive correlations with GSC (**Figure 7**). Therefore, these results suggest that the high sucrose levels in the flag leaf under NTS enhance sucrose translocation to the grains. The sucrose delivered to the grains is subsequently cleaved into uridine diphosphate glucose and fructose by the highly active GSS. This cleavage appears to facilitate starch synthesis and ultimately increases grain yield (Volpicella et al., 2016).

In wheat, the flag leaf plays a central role in sugar metabolism as the primary source organ during the grain filling stage (Price et al., 2004; Bläsing et al., 2006). The flag leaf, being the primary organ of photosynthesis, predominantly produces carbohydrates in the form of sucrose, which is the main transportable form of sugar (Samkumar et al., 2022). It influences the accumulation and distribution of photosynthetic products, which subsequently affects grain development and final yield (Wang et al., 2015). The higher activity of FSPS and FSS enzymes plays a crucial role in promoting grain filling and enhancing yield (Chen et al., 2019; Stein and Granot, 2019). In this study, the NTS treatment enhanced FSS activity. This enhancement facilitated sucrose synthesis and maintained a concentration gradient for phloem loading. Consequently, sucrose was effectively transported to developing grains (Shi et al., 2016).

Path analysis indicated that GSS activity indirectly affects grain yield mainly by cleaving GSC and promoting starch synthesis. This suggests that NTS enhances GSS activity, potentially facilitating the cleavage of sucrose into substrates for starch biosynthesis, thereby accelerating the grain filling process and increasing grain yield (Takeda et al., 2017). Additionally, the FSSC and FSC under T treatment peaked earlier in 2024. This early peak restricted cell division in the endosperm of the grains, ultimately resulting in smaller grain size and reduced grain yield (Girousse et al., 2018; Ahmed et al., 2020). This suggests that an earlier peak sugar content is associated with a reduction in grain yield. From an agronomic perspective, thousand-grain weight showed no significant differences among treatments (**Table 4**). However, under NTS treatment, spike count and grains per spike increased significantly. These increases were the main contributing factors to grain yield improvement. These results indicate that NTS primarily enhances grain yield by promoting spike formation and grain setting rate rather than by increasing grain weight, thereby effectively improving the overall WUE of wheat.

## 5 Conclusion

On the Loess Plateau, long-term NTS improves the grain yield and WUE of wheat. This improvement is achieved through a cascade pathway of “water – sugar metabolism – grain filling”. Sugar metabolism serves as a central hub, linking improved soil water conditions to increased grain yield and enhanced WUE. NTS enhances soil water retention capacity and improves flag leaf water status. Improved flag leaf water status enhances the activities of FSPS and FSS, this promotes sucrose synthesis and its efficient transport to grains. In the grains, highly active SS catalyzes the conversion of sucrose into uridine diphosphate glucose and fructose, providing substrates for starch synthesis. This facilitates grain filling and starch accumulation, ultimately increasing grain yield and WUE.

## Data Availability

The original contributions presented in the study are included in the article/supplementary material. Further inquiries can be directed to the corresponding author.
